# New Breeding Technologies in Grasses

**DOI:** 10.3390/ijms24087295

**Published:** 2023-04-14

**Authors:** Agata Gadaleta, Jose Miguel Soriano

**Affiliations:** 1 Department of Soil, Plant and Food Science, University of Bari Aldo Moro, 70126 Bari, Italy; 2 Sustainable Field Crops Programme, IRTA (Institute for Food and Agricultural Research and Technology), 25198 Lleida, Spain

Plant breeding is continuously evolving to develop new cultivars with the desired traits in the most efficient way. This progress has been particularly significant from the 1980s, when agriculture and plant breeding experienced a great impulse due to the development of molecular biology. This advancement allowed plant scientists and breeders to understand the genetic basis of complex traits, leading to their manipulation to produce improved cultivars. New plant-breeding techniques (NBT) report economic advantages as the time to obtain a new cultivar is reduced. Overall, the NBT permits breeders to introduce a target trait in an efficient and precise way, thus accelerating the selection procedure. This Special Issue focuses on recent advances in “New Breeding Technologies in Grasses”, with novel research and reviews, which cover all related topics, including new marker technologies, fine mapping and gene discovery, and speed breeding. This issue will present a good picture of the state-of-the-art and future potential of breeding approaches.

This Special Issue includes a total of five published papers, of which four were original research manuscripts using different mapping and sequencing techniques, and the last one makes a review of the genomic selection to restore plant material.

In the last years, especially with the development of high-throughput genotyping techniques, the Genome Wide Association Study (GWAS) has become a very powerful methodology to dissect traits of interest in crops. The use of reference maps or genomes, instead of developing new ones, as well as the high number of recombination events among germplasm collections, allow fine mapping in a more efficient way, especially when used in combination with the meta-analysis as reported by [1,2] for the identification of QTL (quantitative trait loci) hotspots related to different traits in durum wheat, enhancing the detection of candidate genes useful for positional cloning.

Yannam et al. [3] summarized the use of genome-wide association analysis for phenology, climatic data, and differentiation patterns among wheat Mediterranean landraces. The analysis led to the identification of 651 marker-trait associations grouped in 46 QTL hotspots. The authors used a new approach, based on long-term climatic data from the regions of origin of the landraces, in combination with genomic analyses for the detection of genome regions, controlling traits involved in the adaptation to environmental conditions. This approach is called environmental (env) GWAS, and its importance resides in correlating these genomic regions with yield-related traits, being that abiotic stress strongly affects yield-related traits in all cereals [4]. Moreover, the authors used eigenvectors to understand the genetic differences among wheat Mediterranean landraces, and, out of the eleven QTL hotspots, reporting significant associations by eigen GWAS, six hotspots on chromosomes 2B, 5B, 6B, 7A, and 7B showed allelic or haplotype differences among genetic subpopulations, thus considering them as the main drivers of genetic differentiation among Mediterranean landraces.

Liu et al. [5] used another important NBT, such as transcriptome sequencing, to mine candidate genes under low-temperature stress and other abiotic stresses in order to understand the molecular mechanism of centipede grass in response to low-temperature stress in maize, and they measured physiological indicators and sequenced the transcriptome of centipede grass under different stress durations. Eight hundred and eighty-five differentially expressed transcripts were obtained, and, through the KEGG (the Kyoto Encyclopedia of Genes and Genomes) enrichment analysis, they determined that arginine and proline metabolism, plant circadian rhythm, plant hormone signal transduction, and the flavonoid biosynthesis pathways played important roles in the cold stress resistance of centipede grass. In addition, by using weighted gene co-expression network analysis (WGCNA), the turquoise module was found to be significantly correlated with soluble sugar (SS) content and ascorbic acid peroxidase (APX) activity.

A GWAS was also performed by Yannam et al. [6] in maize by using 400 inbred lines with 156,164 SNPs to study the genetic architecture of senescence-related traits and their relationship with agronomic traits. They estimated the timing of senescence to be 45 days after anthesis in the whole plant and specifically in the husks. A list of genes was confirmed to be involved in senescence (core senescence genes). Eight significant single nucleotide polymorphisms, SNPs, were found located in the coding or promoter regions of core senescence genes identified in a previous RNAseq experiment. One of the candidate genes, Zm00001d026501, codifies the plastidic glutamine synthetase 2 enzyme (GS2), which is involved in the assimilation of photorespiratory ammonium, as reported by several authors, and, in different species, these results work together with other genes [7,8,9].

The use of new source of alleles for improving wheat is an efficient strategy in plant breeding, above all for yield and quality, as it is considered a prerequisite to obtain new cultivars.

Grain dietary fiber content is an important health-promoting trait of bread wheat. A dominant dietary fiber component of wheat is the cell wall, polysaccharide arabinoxylan, and the goat grass, *Aegilops biuncialis,* which has high β-glucan content, aand this makes it an attractive gene source to develop wheat lines with modified fiber composition [10,11]. In a paper by Ivanizs et al. [10], which investigated the support of introgression breeding, genetic variability in grains’ β-glucan, pentosan, and protein content was conducted in a collection of *A. biuncialis*. A large variation in grain protein and edible fiber content was revealed, reflecting the origin of *A. biuncialis* accessions from different eco-geographical habitats. Association analysis, using DArTseq-derived SNPs, identified 34 QTLs associated with β-glucan, pentosan, water-extractable pentosan, and protein content. Mapping the markers to draft chromosome assemblies of diploid progenitors of *A. biuncialis* underlined the role of genes on chromosomes 1M^b^, 4M^b^, and 5M^b^ in the formation of grain β-glucan content, while other QTLs on chromosome groups 3, 6, and 1 identified the genes responsible for total- and water-extractable pentosan content.

The last paper in the collection is a review, summarizing the use of genomic selection to develop performance-based restoration plant materials [12]. Jones et al. illustrate a multi-disciplinary approach, based on genomic selection, to develop plant materials that address environmental issues constraining local populations in altered ecosystems. Based on DNA sequencing and genomic selection, it is possible to develop a rapid screening of large numbers of seedlings, even for traits expressed only in more mature plants. Plants are genotyped and phenotyped in a training population to develop a genome model for the desired phenotype. Populations with modified phenotypes can be used to identify plant syndromes and test basic hypotheses regarding relationships of traits to adaptation and to one another. The effectiveness of genomic selection in crop and livestock breeding suggests this approach has tremendous potential for improving restoration outcomes for species, such as blue bunch wheatgrass.

This present Special Issue was very successful and demonstrated new breeding technologies, here described, which are very efficient in order to study a number of traits, form yield to quality, in a climate change condition.
